# TSUBASA study: evaluation of the quality and content of daily life of people with hemophilia A without factor VIII inhibitors on prophylactic treatment with emicizumab

**DOI:** 10.1016/j.rpth.2025.102971

**Published:** 2025-07-16

**Authors:** Teruhisa Fujii, Keiji Nogami, Akihiro Sawada, Azusa Nagao, Chiai Nagae, Masanori Nojima, Nobuaki Suzuki, Mika Kawano, Tomomi Shimura, Yoshimasa Sugao, Kagehiro Amano

**Affiliations:** 1 Division of Transfusion Medicine, Hemophilia Treatment Center, Hiroshima University Hospital, Hiroshima, Japan; 2 Department of Pediatrics, Nara Medical University, Nara, Japan; 3 Department of Respiratory Medicine and Hematology, Hyogo Medical University, Hyogo, Japan; 4 Department of Hematology and Oncology, Kansai Medical University Hospital, Osaka, Japan; 5 Department of Pediatrics, St. Marianna University School of Medicine, Kanagawa, Japan; 6 Division of Advanced Medicine Promotion/Center for Translational Research, Institute of Medical Science, University of Tokyo, Tokyo, Japan; 7 Department of Transfusion Medicine, Nagoya University Hospital, Aichi, Japan; 8 Chugai Pharmaceutical Co, Ltd, Tokyo, Japan; 9 Department of Laboratory Medicine, Tokyo Medical University, Tokyo, Japan

**Keywords:** hemophilia, emicizumab, Japan, prospective study, quality of life

## Abstract

**Background:**

Hemophilia A (HA) negatively impacts quality of life (QoL). Treatment with the bispecific antibody emicizumab has shown efficacy and safety in people with HA in clinical trials, but long-term QoL data are limited.

**Objectives:**

To investigate the QoL of people with HA receiving emicizumab over 97 weeks in the prospective, observational TSUBASA study in Japan.

**Methods:**

Data were collected from participants aged ≥6 years with HA without factor VIII inhibitors and caregivers of participants of any age from November 2019 to October 2023. Quality of daily life was measured via the 36-Item Short Form Health Survey, the International Physical Activity Questionnaire, the Work Productivity and Activity Impairment Questionnaire and Classroom Impairment Questionnaire: Hemophilia Specific, and a survey-based questionnaire evaluating daily life, completed by the participant and their caregiver.

**Results:**

Overall, 104 participants aged ≥6 years were enrolled. The median (range) age was 39.0 (6-73) years. Eighty-five (81.7%) participants had severe HA; 21 (20.2%) had target joints. The 36-Item Short Form Health Survey scores were mostly unchanged across the study period and comparable with Japanese national standard values. The proportion of participants engaging in high physical activity increased from 20.2% to 27.3% between baseline and week 97. The Work Productivity and Activity Impairment Questionnaire and Classroom Impairment Questionnaire: Hemophilia Specific scores were generally stable. The questionnaires showed improvements in activity, motivation for work/school, and bleed anxiety, as judged by participants and caregivers.

**Conclusion:**

QoL outcomes remained largely unchanged across the study period. Notable improvements were observed in physical activity levels, motivation for work/school, and anxiety related to bleeding, as reported by participants and caregivers.

## Essentials

• Real-world emicizumab QoL data are limited in people with HA.
• In the observational TSUBASA study, QoL of people with HA initiating emicizumab was measured over 97 weeks.
• QoL outcomes remained largely unchanged across the study period.
• Participants reported improvements in physical activity, work motivation, and bleeding anxiety.

## Visual Abstract

**Figure:**
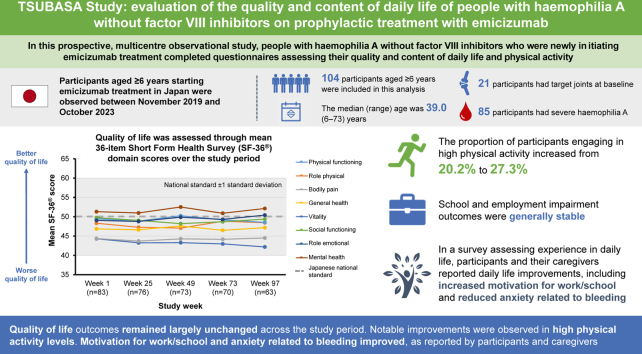

## 1 Introduction

Congenital hemophilia A (HA) is characterized by a lack of endogenous factor (F)VIII [1], which leads to a tendency for bleeding, predominantly into the joints, potentially causing joint pain and functional impairment [2]. This can negatively impact participation in physical and social activities and affect engagement with school and employment [[3], [4], [5]], which may be compounded by a fear of bleeding associated with physical activities [6]. In addition to day-to-day concerns related to the potential for bleeding events to occur, this can impact the quality of life (QoL) and psychosocial health of those affected and their caregivers [1,3,5,7].

The World Federation of Hemophilia and the International Society on Thrombosis and Haemostasis recommend regular administration of hemostatic agents [1,8], the aim of which is to prevent bleeding and allow people with HA to live active lives and reach a QoL comparable with those without hemophilia [1]. Although prophylactic FVIII replacement offers clinical benefit, most treatment options have short half-lives that necessitate intravenous infusions multiple times per week [9]. This represents a significant treatment burden and has demonstrated association with poor QoL in people with HA [5].

Emicizumab, a recombinant, bispecific monoclonal antibody, mimics the function of activated FVIII by bridging activated FIX and FX [10,11]. It is approved for the treatment of people with HA of all ages with or without FVIII inhibitors [12], and has demonstrated effective hemostasis and a tolerable safety profile in many clinical trials [[13], [14], [15], [16], [17]]. Emicizumab is administered subcutaneously at 1.5 mg/kg weekly, 3 mg/kg every 2 weeks, or 6 mg/kg every 4 weeks, which may reduce the treatment burden compared with FVIII prophylaxis. The sustained plasma drug concentrations reported with emicizumab [18] may also reduce bleeding anxiety compared with FVIII prophylaxis. Clinical trials have shown positive QoL outcomes in people with HA after initiating emicizumab treatment [15,19,20]; however, real-world, long-term QoL data are limited.

The TSUBASA study (UMIN-CTR-ID: UMIN0000377448) was designed to explore the relationship between physical activity and bleeding events and assess safety and QoL in a Japanese cohort of people with HA initiating emicizumab prophylaxis. Here, we report an evaluation of the quality and content of daily life and physical activity among people with HA on prophylactic emicizumab treatment in the TSUBASA study.

## 2 Methods

### 2.1 Study design and participants

TSUBASA is a prospective, multicenter, observational study conducted across 50 participating institutions in Japan; the study design details have been published previously [21,22]. Eligible study participants were planned to begin emicizumab treatment and had a diagnosis of congenital HA without FVIII inhibitors at the time of enrolment; emicizumab was selected as the most appropriate treatment after investigator evaluation. Participants previously treated with emicizumab were excluded to facilitate assessment of changes in QoL after initiating treatment. Participants must have received emicizumab at least once during the study and responded to one or more of the assessment questionnaires to be included in this final analysis.

For the current analysis, participants aged <6 years were excluded. These participants are the focus of a separate analysis of the TSUBASA study. A questionnaire-based survey was completed by participants ≥6 years old and their caregivers of all ages.

The study was conducted in compliance with the International Conference on Harmonization Guidelines for Good Clinical Practice, the Ethical Guidelines for Medical and Health Research Involving Human Subjects, and the Declaration of Helsinki. The study protocol was approved by the research ethics committee at each study site.

Written informed consent was provided by all participants or their legally acceptable representatives before entering the study.

### 2.2 Endpoints

General QoL was measured using the 36-Item Short Form Health Survey (SF-36), in which participants answered 36 questions across 8 domains [23]. The SF-36 was completed by study participants aged ≥16 years (Supplementary Table 1).

The International Physical Activity Questionnaire (IPAQ) was used to estimate mean weekly activity intensity per participant (classified as low, moderate, or high activity) [24]. The IPAQ was completed by participants aged ≥6 years.

The participants’ employment or schooling status was evaluated through the Work Productivity and Activity Impairment Questionnaire and Classroom Impairment Questionnaire: Hemophilia Specific (WPAI+CIQ:HS), which quantifies work/school absence, productivity, and impairment within the 7 days preceding questionnaire completion [25]. The WPAI+CIQ:HS was completed by participants aged ≥6 years.

A survey-based questionnaire was used to evaluate the daily life of participants, completed by participants aged ≥6 years or caregivers of participants of any age, to quantify perceived improvements from baseline. A section for any additional comments was also included in the questionnaire.

### 2.3 Data collection

Details on data collection have been published previously [21]. All participant- and caregiver-reported data were collected using a mobile device application (electronic patient-reported outcome). Questionnaires were completed at weeks 1, 49, and 97.

### 2.4 Statistical analysis

No confirmatory hypotheses were established as the study is descriptive. The endpoints were summarized using descriptive statistics and represented as means, with changes from baseline to week 97. For the SF-36, participants were given a score of 0–100, depending on their answers to the questionnaire (100 representing the highest QoL). SF-36 scores were presented against the 2017 Japanese national population standard, set out by i-Hope International (Kyoto, Japan), which was represented by a score of 50 (SD, 10).

Analyses were also performed in the following subgroups: on-demand or prophylactic FVIII therapy at baseline, HA severity (severe or moderate), presence/absence of target joints, and bleeding history at enrolment. For each SF-36 domain, participants scoring ≤40 were categorized by the presence of baseline comorbidities.

## 3 Results and Discussion

In the TSUBASA study, 129 male participants were enrolled between November 1, 2019, and October 31, 2021. Of these, 104 were aged ≥6 years and were included in this analysis (Table 1). The median (minimum-maximum) age of the participants was 39.0 (6-73) years. Overall, 21 (20.2%) participants had ≥1 target joint at baseline; 65 (62.5%) had ≥1 comorbidity. In total, 103 (99.0%) participants reported use of FVIII replacement products prior to study entry.

**Table 1:** Baseline demographics in the overall study population and analysis population (participants aged ≥6 years).

Characteristic	Analysis population (*N* = 104) *n* (%)
Age, y	
Median (min-max)	39.0 (6-73)
Age group (y)	
<2	−
2-5	−
6-11	12 (11.5)
12-17	9 (8.7)
18-39	35 (33.7)
40-64	41 (39.4)
≥65	7 (6.7)
Hemophilia severity	
Severe	85 (81.7)
Moderate	19 (18.3)
Report of bleeding in 24 wk prior to study entry	
None	34 (32.7)
Yes	65 (62.5)
Unknown	5 (4.8)
Use of coagulation FVIII products prior to study entry	
None	1 (1.0)
Present	103 (99.0)
Prophylaxis	82 (79.6)
On-demand	21 (20.4)
History of immune tolerance induction therapy	
None	99 (95.2)
Present	4 (3.8)
Unknown	1 (1.0)
Target joint^a^	
None	83 (79.8)
Present	21 (20.2)
1 target joint	11 (10.6)
2 target joints	8 (7.7)
≥3 target joints	2 (1.9)
Comorbidities^b^	
None	39 (37.5)
1-2	36 (34.6)
≥3	29 (27.9)

FVIII, factor VIII; HIV, human immunodeficiency virus; Max, maximum; Min, minimum.

a Defined as joints with ≥3 bleeds occurring over the 24 weeks prior to study entry.

b The most common comorbidities included hypertension (20 cases), hepatitis C infection (16 cases), hemophilic arthropathy (9 cases), and HIV infection (9 cases).

**Figure 1:**
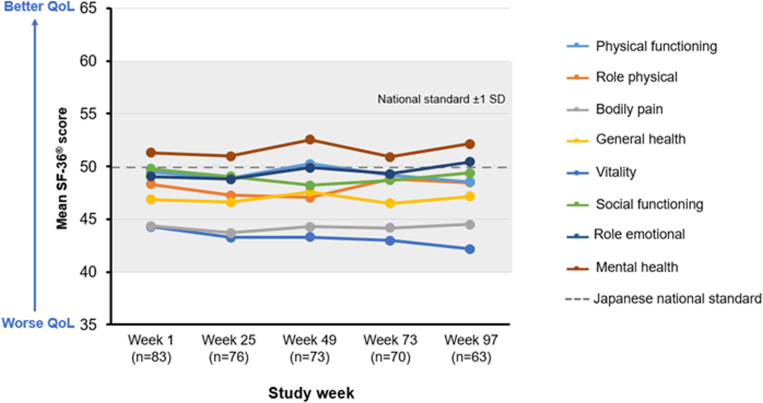
36-Item Short Form Health Survey (SF-36) domain scores over the study period. QoL, quality of life.

Eighty-three participants aged ≥16 years completed the SF-36 at baseline (Figure 1). The SF-36 domain scores remained consistent across the 2-year study period and were mostly comparable with Japanese national standard values. “Bodily pain” and “physical functioning” were the lowest scoring, with respective mean scores of 44.50 and 42.18 at week 97 (*n* = 63). Scores for “mental health” and “role emotional” were 52.13 and 50.42, respectively, both exceeding the Japanese national standard. Subgroup analyses are presented in Supplementary Figure 1 and Supplementary Table 2.

SF-36 scores have not previously been used to evaluate overall QoL during emicizumab treatment. However, SF-36 scores for the general HA population have demonstrated worse outcomes in physical domains compared with the population without HA [26], and lower scores in all domains in another report [27]. “Physical functioning” and “bodily pain” scored the lowest in this study in comparison with the national standard, but other domains reflected improved HA care since the publication of previous reports [26,27]. At baseline, 20.2% of participants had target joints, potentially contributing to lower scores in the “physical functioning” and “bodily pain” domains. Further, of 23 participants scoring 40 or lower in the “physical functioning” domain at baseline, 14 (60.9%) had ≥3 comorbidities, 5 (21.7%) had 1–2 comorbidities, and only 4 (17.4%) had none. Although less severely impacted than the physical domains, the SF-36 mental health domains have also shown lower scores compared with the population without HA [26,28]. The SF-36 “mental health” and “role emotional” outcomes reported here do not follow this trend.

**Figure 2:**
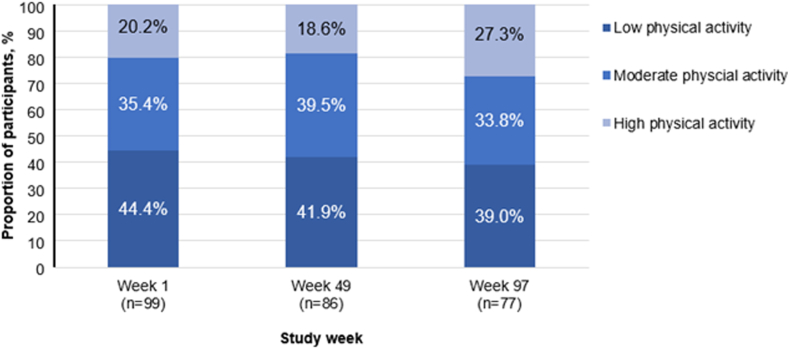
International Physical Activity Questionnaire scores over the study period.

IPAQ scores indicated that 44 of 99 (44.4%), 36 of 86 (41.9%), and 30 of 77 (39.0%) participants reported low physical activity in weeks 1, 49, and 97, respectively (Figure 2). The number of participants reporting high physical activity decreased from 20 of 99 (20.2%) at week 1 to 16 of 86 (18.6%) at week 49, then increased to 21 of 77 (27.3%) at week 97. IPAQ subgroup analyses are presented in Supplementary Figure 2. There are limited studies reporting on the association of emicizumab treatment and physical activity. One cohort study, carried out on pediatric people with HA, found that physical activity increased following emicizumab initiation, as reflected by an increase in the Median Paediatric Haemophilia Activities List sum score from 59.5 at baseline to 84.0 with emicizumab (*P* < .001) [29]. Additionally, in the HAVEN 6 clinical trial, participants treated with emicizumab demonstrated consistent levels of moderate to vigorous physical activity, increasing from approximately 100 daily minutes at baseline to approximately 115 daily minutes at week 25 [30].

WPAI+CIQ:HS scores typically remained stable over the 2-year study period (Table 2). The largest improvement was observed in the percentage of impairment in the classroom due to hemophilia, which decreased from a mean (SD) proportion of 11.0% (16.1) at baseline (*n* = 21) to 8.2% (15.9) at week 97 (*n* = 17). The mean (SD) percentage of overall work impairment increased from 13.7% (19.0) at baseline (*n* = 59) to 18.6% (25.8) at week 97 (*n* = 46). Subgroup analyses of WPAI+CIQ:HS scores are presented in Supplementary Tables 3 and 4.

**Table 2:** Work Productivity and Activity Impairment Questionnaire and Classroom Impairment Questionnaire: Hemophilia Specific scores in the study population.

	Wk 1	Wk 25	Wk 49	Wk 73	Wk 97
Percentage of work time missed due to hemophilia					
No. of participants	59	52	52	52	46
Mean (SD)	1.2 (4.9)	1.6 (5.1)	0.9 (5.6)	1.9 (8.9)	6.1 (20.9)
Min-max	0.0-28.0	0.0-25.0	0.0-40.0	0.0-60.0	0.0-100.0
Percentage of impairment while working due to hemophilia					
No. of participants	59	52	53	53	46
Mean (SD)	12.9 (17.9)	13.1 (20.0)	11.9 (21.9)	13.8 (18.3)	13.5 (18.2)
Min-max	0.0-80.0	0.0-80.0	0.0-100.0	0.0-80.0	0.0-70.0
Percentage of overall work impairment due to hemophilia (work time missed + impairment while working)					
No. of participants	59	52	52	52	46
Mean (SD)	13.7 (19.0)	14.1 (21.0)	10.4 (18.9)	15.1 (20.4)	18.6 (25.8)
Min-max	0.0-80.0	0.0-82.0	0.0-88.0	0.0-84.0	0.0-100.0
Percentage of class time missed due to hemophilia					
No. of participants	20	19	16	16	17
Mean (SD)	0.6 (2.7)	2.1 (6.3)	5.2 (20.0)	2.2 (8.6)	3.3 (10.2)
Min-max	0.0-12.0	0.0-20.0	0.0-80.0	0.0-34.5	0.0-40.0
Percentage of impairment in the classroom due to hemophilia					
No. of participants	21	19	16	16	17
Mean (SD)	11.0 (16.1)	5.8 (11.7)	7.5 (20.2)	6.3 (10.9)	8.2 (15.9)
Min-max	0.0-50.0	0.0-40.0	0.0-80.0	0.0-30.0	0.0-50.0
Percentage of overall classroom impairment due to hemophilia (class time missed + impairment in the class)					
No. of participants	20	19	16	16	17
Mean (SD)	12.1 (16.1)	7.5 (14.0)	11.7 (27.7)	8.2 (13.9)	11.5 (17.3)
Min-max	0.0-50.0	0.0-52.0	0.0-84.0	0.0-41.0	0.0-50.0
Percentage of activity impairment due to hemophilia					
No. of participants	100	95	91	86	81
Mean (SD)	21.7 (23.8)	22.6 (26.9)	20.4 (26.4)	22.4 (26.8)	19.5 (23.2)
Min-max	0.0-100.0	0.0-100.0	0.0-100.0	0.0-100.0	0.0-80.0

Max, maximum; Min, minimum.

Baseline WPAI+CIQ:HS scores were generally lower than those reported in other studies [31,32]. Previous trials have shown improvements in employment and schooling for people with HA following emicizumab initiation [7,17,19]. In a pooled analysis of the HAVEN 3 and 4 clinical trials, participants reporting no missed workdays in a 28-day period increased from 75% at baseline to 91% at week 73 of emicizumab treatment [19]. Contrastingly, the percentage of missed work time increased in the current study. One reason for this increase in work absenteeism may be the impact of the COVID-19 pandemic across the study period, which is likely to have affected both school and employment of the participants. The data showing participant-reported improved motivation for work and school, despite actual classroom/work impairment, further supports this suggestion.

At baseline, 46 (46.5%) of 99 participants who completed the daily life questionnaire said they may be limited in physical activity, and 62 (62.6%) said they may be restricted in exercise (Supplementary Figure 3A). At week 97 (*n* = 82), 8 (9.8%) participants reported an increase, and 18 (22.0%) reported a slight increase in activity and exercise frequency. Eleven (13.4%) participants reported an increase, and 13 (15.9%) reported a slight increase in motivation for work. For anxiety about bleeding, 22 (26.8%) reported a decrease, and 29 (35.4%) reported a slight decrease (Supplementary Figure 3B). Subgroup comparisons are presented in Supplementary Table 5. Individual comments were also collected from some participants who completed the questionnaire (Supplementary Table 6).

At week 1, 19 (76.0%) of 25 participants were taking part in all physical education activities, 5 (20.0%) participated in activities according to the event, and 1 (4.0%) was not participating at all (Supplementary Figure 3C). At week 97 (*n* = 22), 18 (81.8%) were participating in all activities, 3 (13.6%) were participating according to the event, and 1 (4.5%) was not participating at all.

Of 44 caregivers responding at week 1, 12 (27.3%) reported that the participant they care for may be restricted in daily activities (Supplementary Figure 4A). Fourteen (31.8%) said that the participant they care for may be restricted during work/school. In total, 37 (84.1%) reported a concern for bleeding in the participants they care for. At week 97 (*n* = 35), 8 (22.9%) caregivers reported an increase in activity, and 6 (17.1%) reported a slight increase. No caregivers reported a decrease or slight decrease (Supplementary Figure 4B). For work and other initiatives, 6 (17.1%) caregivers reported an increase in motivation, and 3 (8.6%) reported a slight increase. In total, 8 (22.9%) reported reduced anxiety about bleeding, and 12 (34.3%) reported a slight reduction in anxiety. Subgroup comparisons of questionnaire outcomes are shown in Supplementary Table 7. Individual comments provided by the caregivers were also provided to contextualize the questionnaire responses (Supplementary Table 8).

### 3.1 Limitations

This nonrandomized study was not designed to be statistically powered for QoL endpoints, meaning all results are solely descriptive. The employed outcome measures are subjective [[23], [24], [25]], and the SF-36 method of overall QoL evaluation has not been employed in other studies investigating emicizumab, limiting the comparisons that can be made. Additionally, the SF-36 and IPAQ are not specific to people with HA, although they have been previously used in other HA studies [26,27]. The daily life questionnaire for participants and caregivers has not been validated, and the IPAQ and WPAI+CIQ:HS are not validated for the younger participants included in this study [24,25]. Some participants did not adhere to data entry in the electronic patient-reported outcome application, leading to a large proportion of missing longitudinal data points later in the study, and potential over- or underestimation of the impact on QoL. Further, the characteristics of participants with missing data were not evaluated, meaning that potential associations with specific subgroups could not be determined. The study took place over the COVID-19 pandemic, during which activity restrictions, such as stay-at-home orders, school closures, and event cancellations, were in place. The effect of activity restrictions was not quantified in this study.

## 4 Conclusions

The reported outcomes were varied, and a consistent trend was difficult to define. Improvements were observed in high physical activity participation in the IPAQ, and participant- and caregiver-reported improvements in activity and exercise frequency, in addition to motivation for school/work and reduced bleeding anxiety, were reported. However, SF-36 scores showed no change, and WPAI+CIQ:HS scores worsened in some domains. Despite the challenges of reporting QoL observed presently, further research into long-term QoL outcomes is needed, which will contribute to improving treatment and care for people with HA.
