# Supplementary material for: TSUBASA study: evaluation of the quality and content of daily life of people with hemophilia A without factor VIII inhibitors on prophylactic treatment with emicizumab
Source: Res Pract Thromb Haemost. 2025 Jul 16;9(5):102971. doi: 10.1016/j.rpth.2025.102971 (PMC12356469; doi:10.1016/j.rpth.2025.102971)
Supplement: Supplementary Material [file mmc1.docx]

**SUPPLEMENTARY MATERIAL**


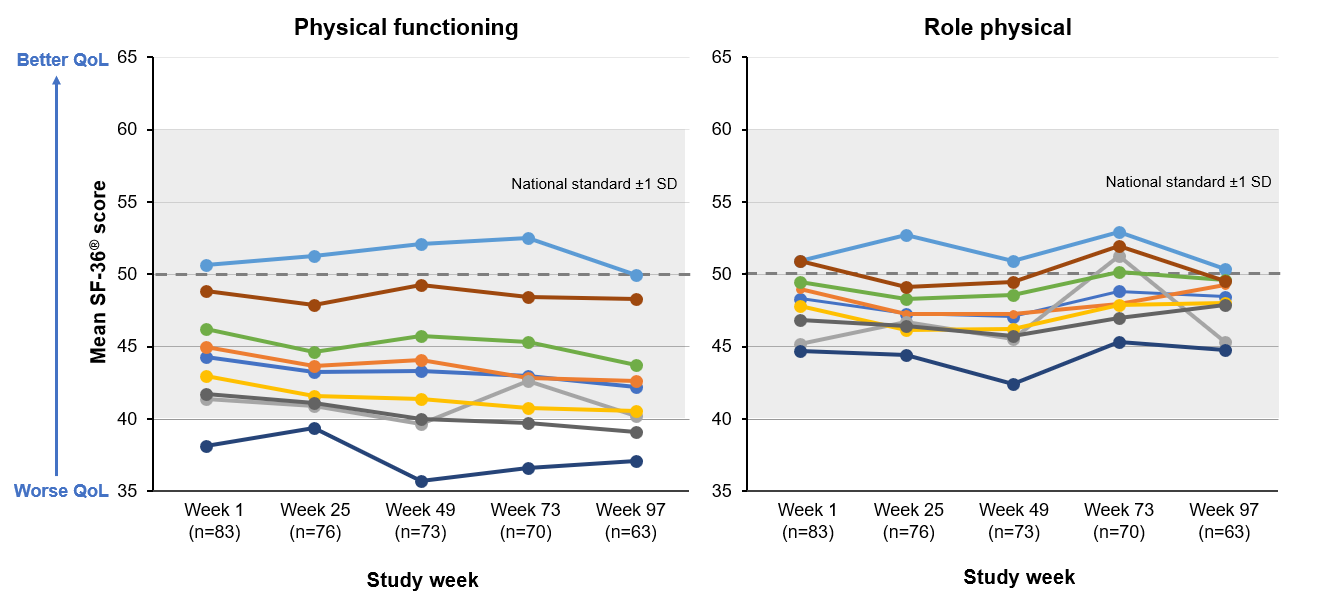
**Figure S1.** SF-36® domain scores by participant subgroups


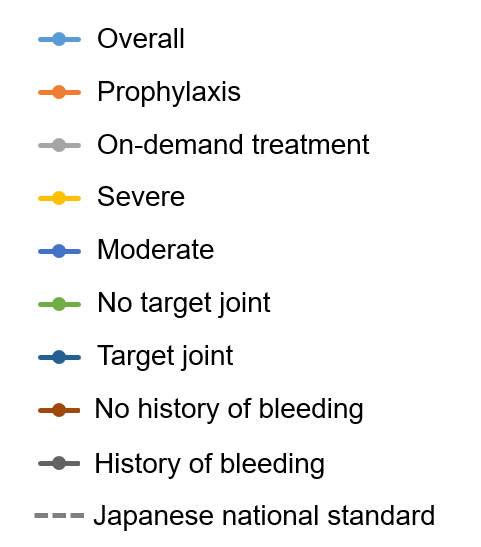


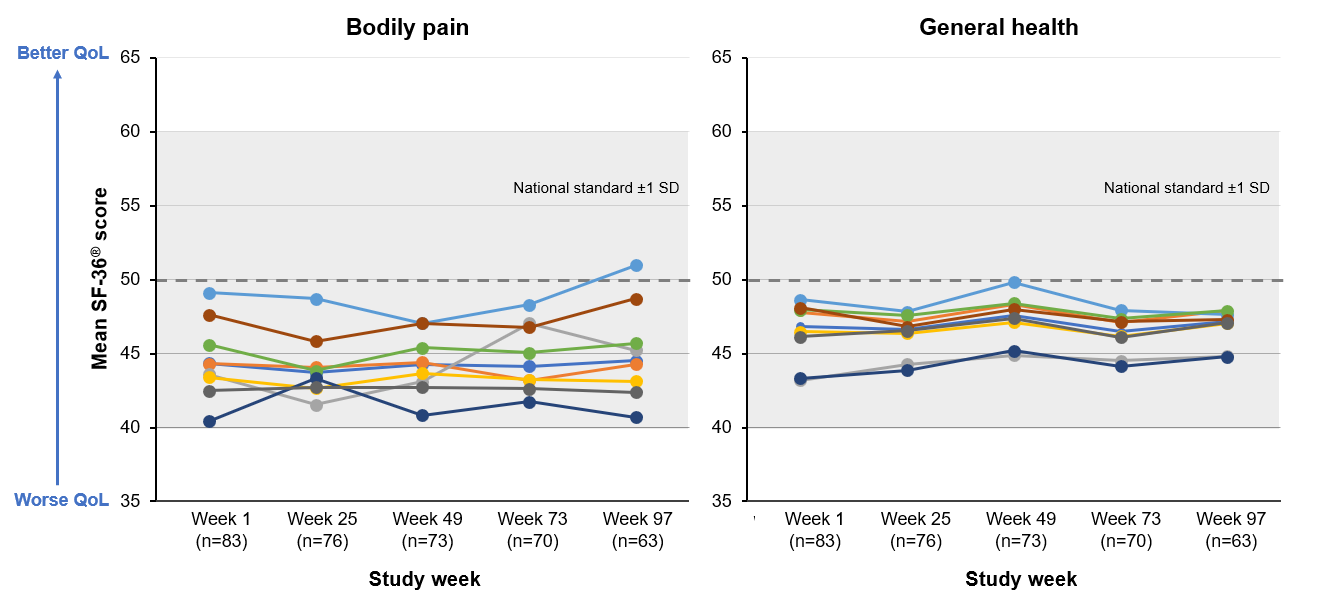


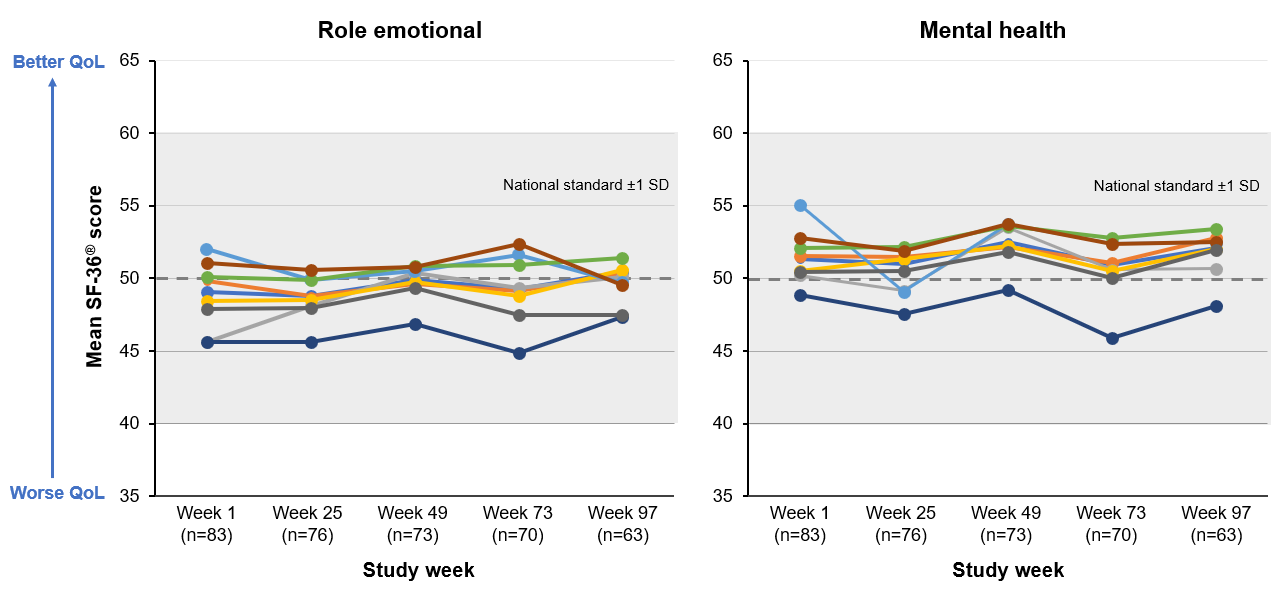
QoL, quality of life; SD,
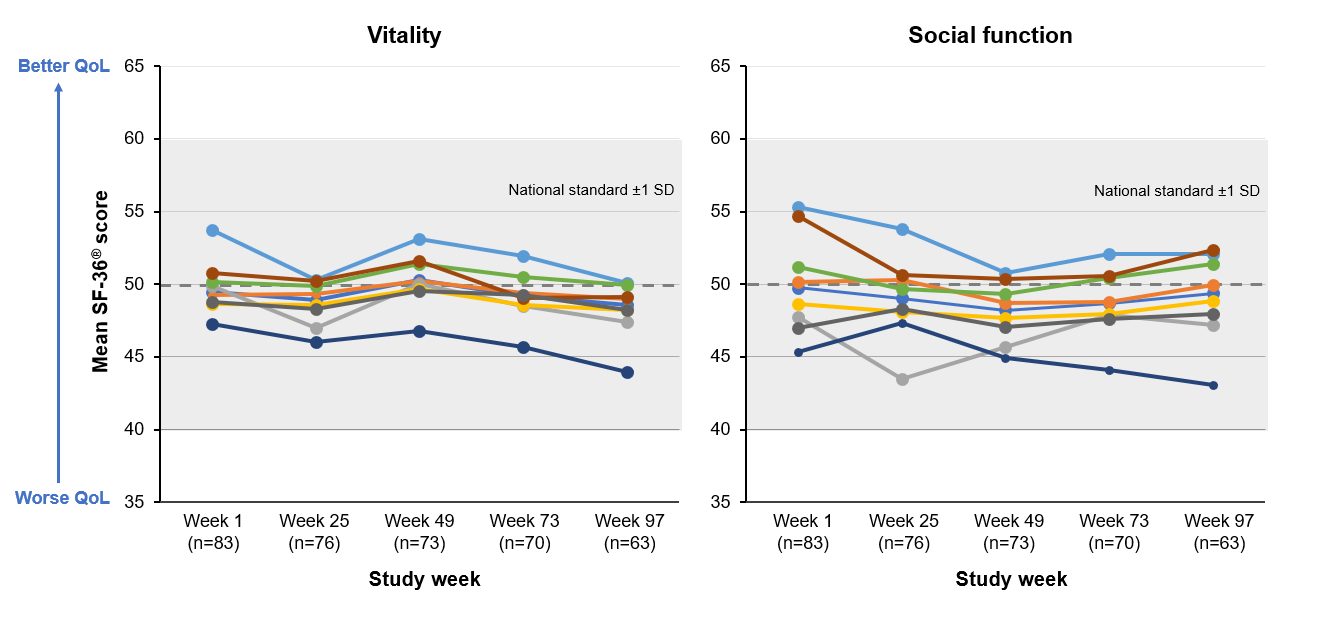
 standard deviation; SF-36®, 36-item Short Form Health Survey

**
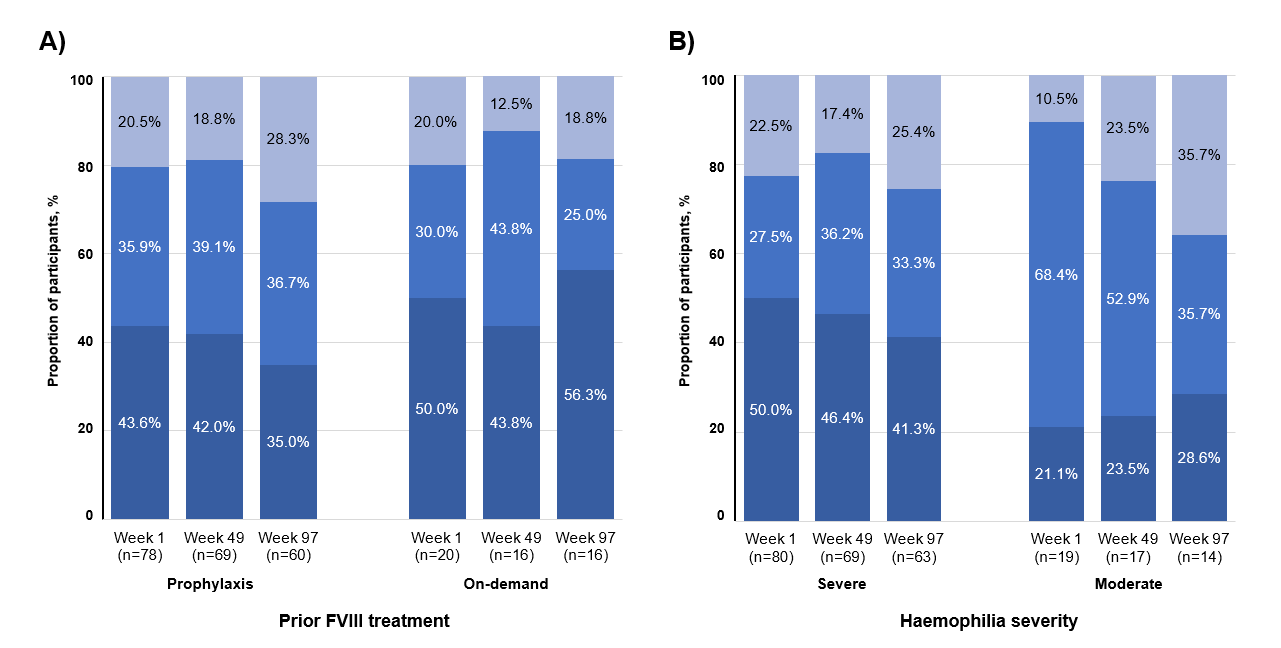
Figure S2.** IPAQ scores by participant subgroup


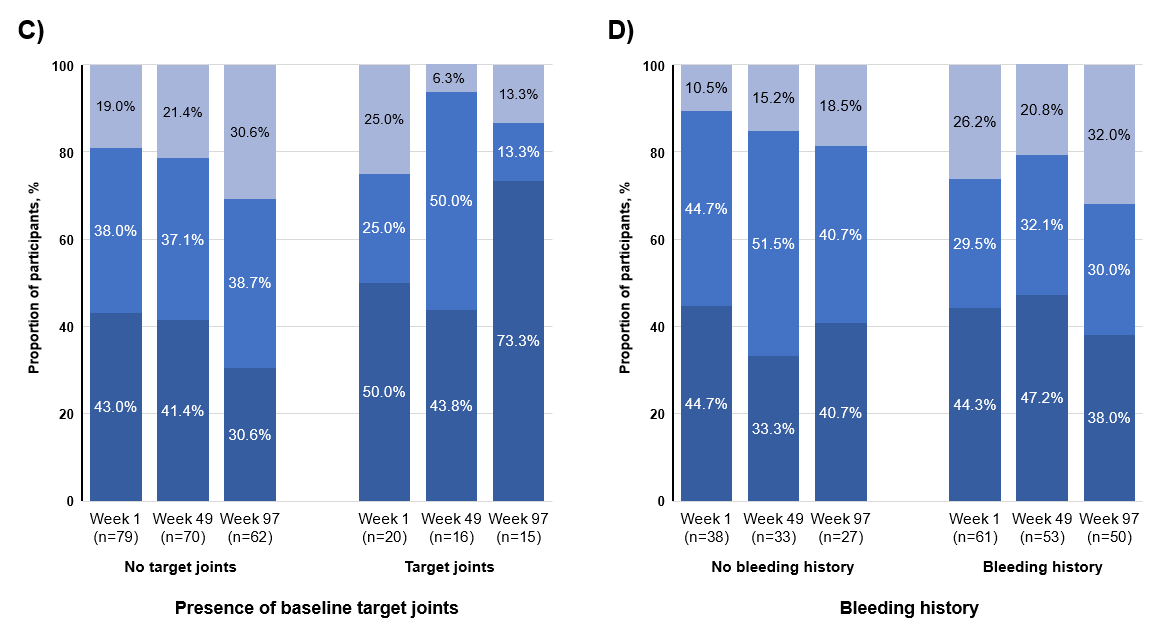


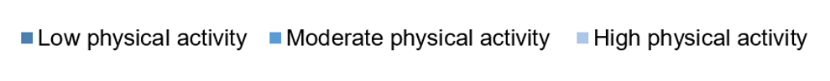


A) Participants on prior prophylactic or on-demand FVIII replacement; B) Participants with severe or moderate HA; C) Participants with or without target joints at baseline; D) Participants with no history of bleeding or history of bleeding.
F, factor; HA, haemophilia A; IPAQ, International Physical Activity Questionnaire


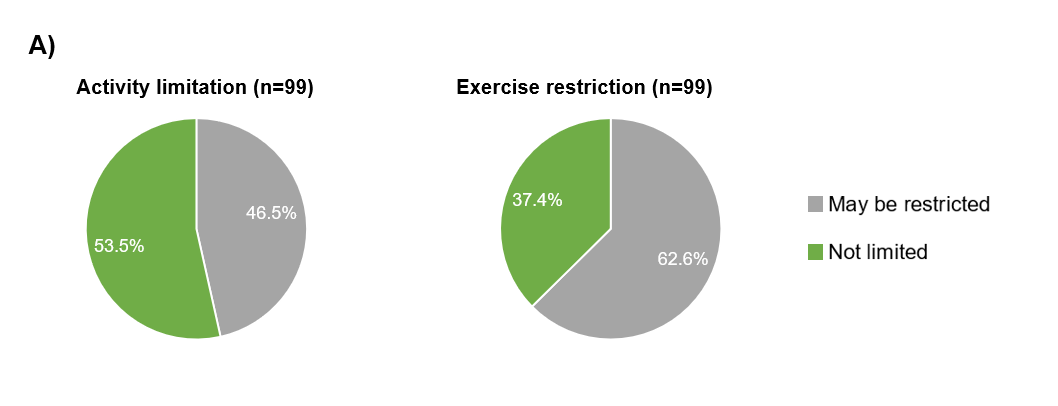
**Figure S3.** Responses from participants in daily life questionnaire


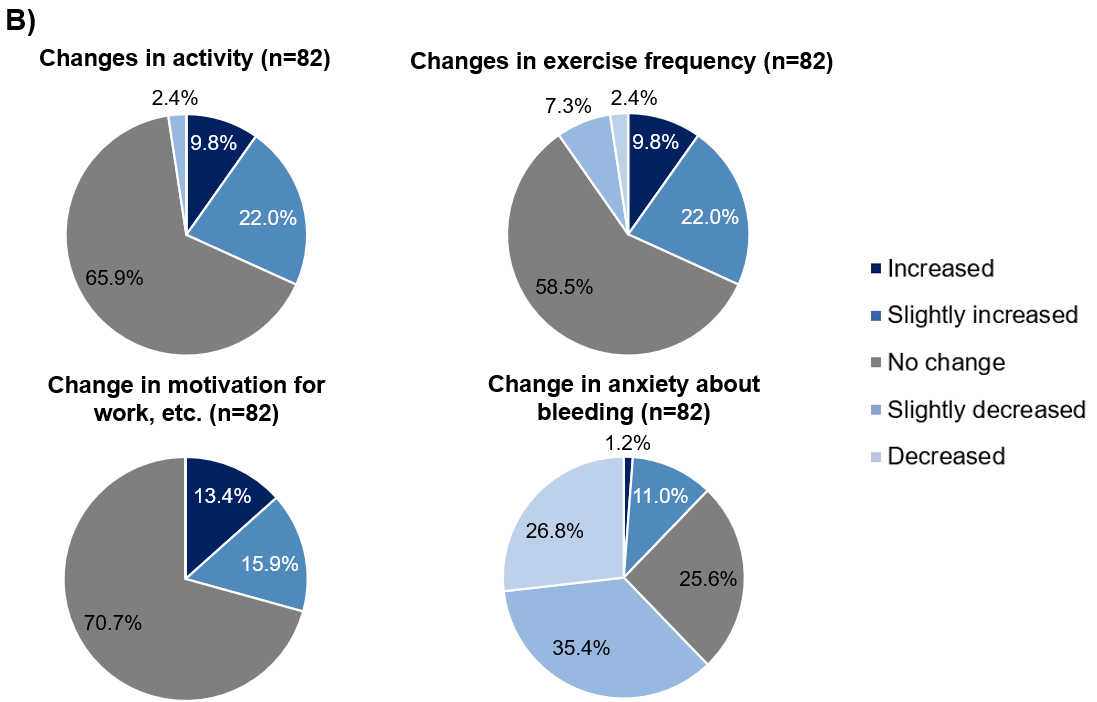


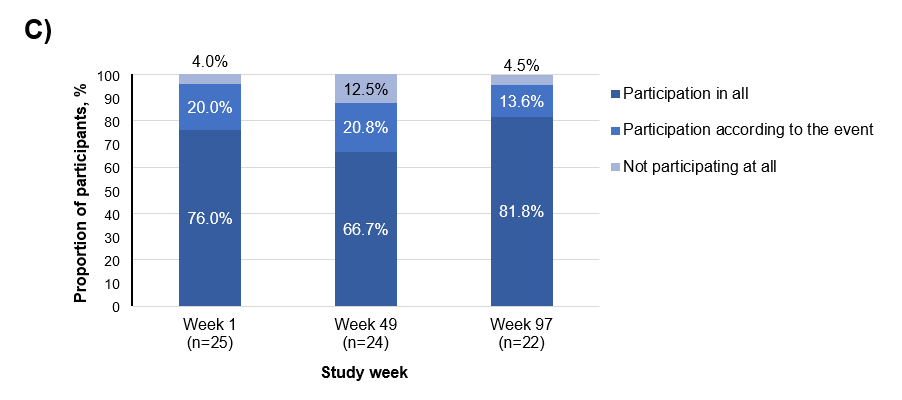


A) Evaluation of participant physical activity at baseline; B) Changes in responses to daily life questionnaire between baseline and Week 97; C) Changes in participation in physical education over the 97-week study period


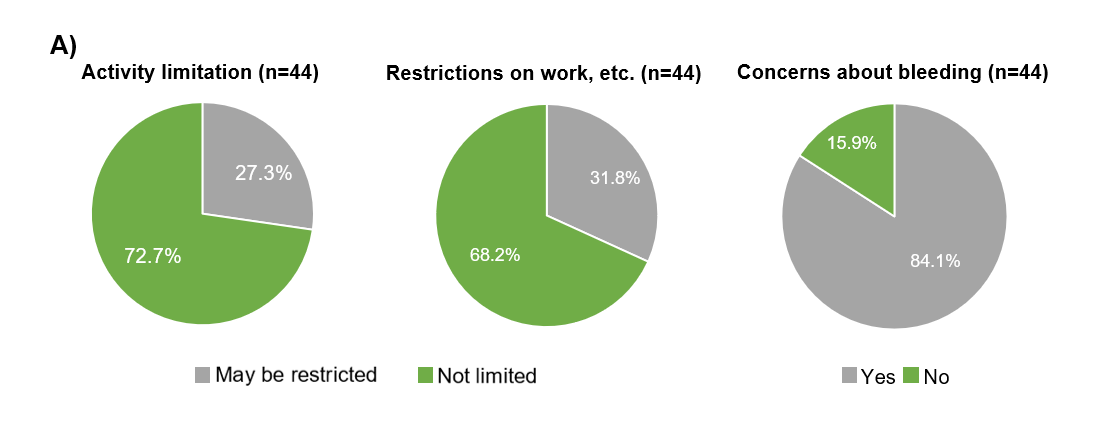
**Figure S4.** Responses from caregivers in the daily life questionnaire


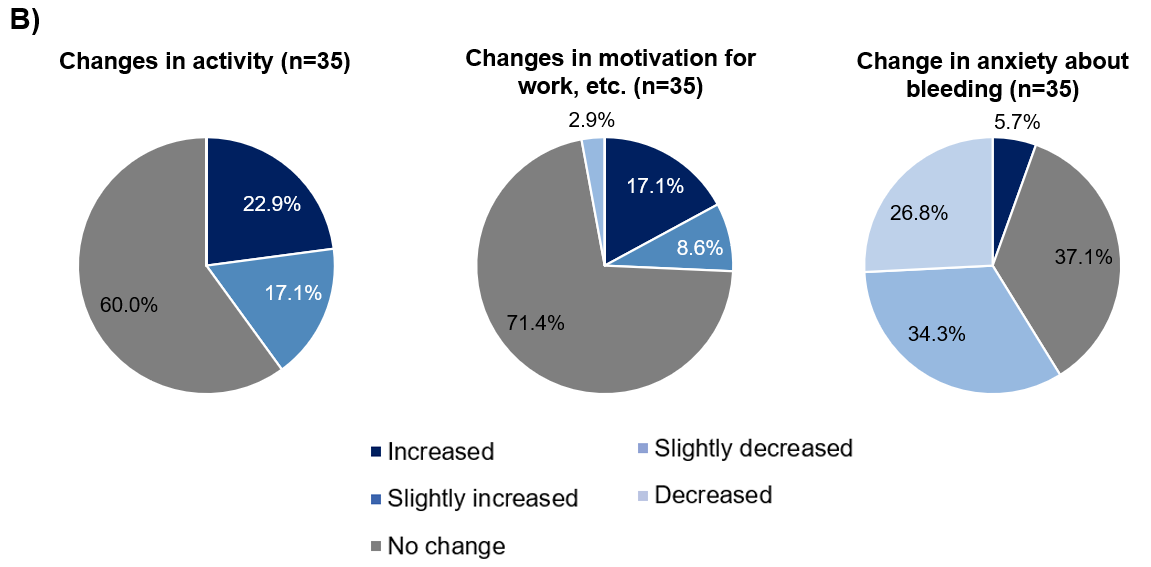


1. Evaluation of participant quality of life at baseline. B) Changes in responses to daily life questionnaire between baseline and Week 97.

**Table S1:** Age requirements for completion of each study questionnaire.

| Study questionnaire | Age for completion |
| --- | --- |
| SF-36® | ≥16 years |
| IPAQ | ≥6 years |
| WPAI+CIQ:HIS | ≥6 years |
| Daily life questionnaire | ≥6 years or caregivers of participants of any age |

IPAQ, International Physical Activity Questionnaire; SF-36®, 36-item Short Form Health Survey; WPAI+CIQ:HS, Work Productivity and Activity Impairment Questionnaire and Classroom Impairment Questionnaire: Hemophilia Specific

**Table S2**: Mean SF-36® domain scores of ≤40 by presence of baseline comorbidities

| SF-36® Domain | Physical functioning | | Role physical | | Bodily pain | | General health | | Vitality | | Social functioning | | Role emotional | | Mental health | | |
| --- | --- | --- | --- | --- | --- | --- | --- | --- | --- | --- | --- | --- | --- | --- | --- | --- | --- |
| Time Point | Week  1 | Week 97 | Week  1 | Week 97 | Week  1 | Week 97 | Week  1 | Week 97 | Week  1 | Week 97 | Week  1 | Week 97 | Week 1 | Week 97 | Week 1 | Week 97 |  |
| Number of subjects | n=83 | n=63 | n=83 | n=63 | n=83 | n=63 | n=83 | n=63 | n=83 | n=63 | n=83 | n=63 | n=83 | n=63 | n=83 | n=63 |  |
| SF-36® score >40,  n (%) | 60 (72.3) | 41 (65.1) | 70 (84.3) | 51 (81.0) | 55 (66.3) | 38 (60.3) | 68 (81.9) | 48 (76.2) | 70 (84.3) | 50 (79.4) | 69 (83.1) | 53 (84.1) | 67 (80.7) | 53 (84.1) | 74 (89.2) | 57 (90.5) |  |
| SF-36® score ≤40,  n (%) | 23 (27.7) | 22 (34.9) | 13 (15.7) | 12 (19.0) | 28 (33.7) | 25 (39.7) | 15 (18.1) | 15 (23.8) | 13 (15.7) | 13 (20.6) | 14 (16.9) | 10 (15.9) | 16 (19.3) | 10 (15.9) | 9 (10.8) | 6 (9.5) |  |
| 0 comorbidities | 4 (17.4) | 3 (13.6) | 2 (15.4) | 4 (33.3) | 4 (14.3) | 6 (24.0) | 2 (13.3) | 3 (20.0) | 3 (23.1) | 5 (38.5) | 2 (14.3) | 2 (20.0) | 1 (6.3) | 4 (40.0) | 2 (22.2) | 2 (33.3) |  |
| 1–2 comorbidities | 5 (21.7) | 8 (36.4) | 4 (30.8) | 1 (8.3) | 8 (28.6) | 9 (36.0) | 3 (20.0) | 4 (26.7) | 2 (15.4) | 4 (30.8) | 6 (42.9) | 4 (40.0) | 5 (31.3) | 2 (20.0) | 1 (11.1) | 2 (33.3) |  |
| ≥3 comorbidities | 14 (60.9) | 11 (50.0) | 7 (53.8) | 7 (58.3) | 16 (57.1) | 10 (40.0) | 10 (66.7) | 8 (53.3) | 8 (61.5) | 4 (30.8) | 6 (42.9) | 4 (40.0) | 10 (62.5) | 4 (40.0) | 6 (66.7) | 2 (33.3) |  |

SF-36®, 36-item Short Form Health Survey

**Table S3.** Subgroup analysis for WPAI+CIQ:HS scores between participants with or without baseline target joints for participants aged ≥6 years

|  | | **No target joint at baseline** | | | | | | | | | **Target joint at baseline** | | | | | | | | | | |
| --- | --- | --- | --- | --- | --- | --- | --- | --- | --- | --- | --- | --- | --- | --- | --- | --- | --- | --- | --- | --- | --- |
|  |  | Week 1 | Week 25 | | Week 49 | | Week 73 | | Week 97 | | Week 1 | | Week 25 | | Week 49 | | Week 73 | | Week 97 | |  |
| Percentage of work time missed due to haemophilia, % | | | | | | | | | | | | | | | | | | | | | |
| Number of subjects | | 45 | | 38 | | 41 | | 39 | | 36 | | 14 | | 14 | | 11 | | 13 | | 10 |  |
|  | Mean (SD) | 1.5 (5.5) | | 1.3 (4.3) | | 0.1 (0.8) | | 0.5 (2.5) | | 4.5 (17.1) | | 0.0 (0.0) | | 2.5 (7.0) | | 3.6 (12.1) | | 6.2 (17.1) | | 12.0 (31.6) |  |
|  | Min–Max | 0.0–28.0 | | 0.0–22.9 | | 0.0–5.4 | | 0.0–15.0 | | 0.0–100.0 | | 0.0–0.0 | | 0.0–25.0 | | 0.0–40.0 | | 0.0–60.0 | | 0.0–100.0 |  |
| Percentage of impairment while working due to haemophilia, % | | | | | | | | | | | | | | | | | | | | | |
| Number of subjects | | 45 | | 38 | | 41 | | 40 | | 36 | | 14 | | 14 | | 12 | | 13 | | 10 |  |
|  | Mean (SD) | 12.7 (19.5) | | 12.9 (21.3) | | 7.3 (11.8) | | 12.3 (14.2) | | 11.9 (16.4) | | 13.6 (12.2) | | 13.6 (16.5) | | 27.5 (37.7) | | 18.5 (27.6) | | 19.0 (23.8) |  |
|  | Min–Max | 0.0–80.0 | | 0.0–80.0 | | 0.0–60.0 | | 0.0–50.0 | | 0.0–70.0 | | 0.0–30.0 | | 0.0–50.0 | | 0.0–100.0 | | 0.0–80.0 | | 0.0–70.0 |  |
| Percentage of overall work impairment due to haemophilia, % | | | | | | | | | | | | | | | | | | | | | |
| Number of subjects | | 45 | | 38 | | 41 | | 39 | | 36 | | 14 | | 14 | | 11 | | 13 | | 10 |  |
|  | Mean (SD) | 13.7 (20.8) | | 13.7 (21.9) | | 7.4 (11.9) | | 12.9 (14.7) | | 15.5 (22.4) | | 13.6 (12.2) | | 15.0 (19.1) | | 21.6 (33.0) | | 21.5 (32.1) | | 29.6 (34.7) |  |
|  | Min–Max | 0.0–80.0 | | 0.0–82.0 | | 0.0–60.0 | | 0.0–50.0 | | 0.0–100.0 | | 0.0–30.0 | | 0.0–62.5 | | 0.0–88.0 | | 0.0–84.0 | | 0.0–100.0 |  |
| Percentage of class time missed due to haemophilia, % | | | | | | | | | | | | | | | | | | | | | |
| Number of subjects | | 20 | | 19 | | 16 | | 16 | | 16 | | 0 | | 0 | | 0 | | 0 | | 1 |  |
|  | Mean (SD) | 0.6 (2.7) | | 2.1 (6.3) | | 5.2 (20.0) | | 2.2 (8.6) | | 3.5 (10.5) | | - | | - | | - | | - | | 0.0 (NE) |  |
|  | Min–Max | 0.0–12.0 | | 0.0–20.0 | | 0.0–80.0 | | 0.0–34.5 | | 0.0–40.0 | | - | | - | | - | | - | | 0.0–0.0 |  |
| Percentage of impairment in the classroom due to haemophilia, % | | | | | | | | | | | | | | | | | | | | | |
| Number of subjects | | 20 | | 19 | | 16 | | 16 | | 16 | | 1 | | 0 | | 0 | | 0 | | 1 |  |
|  | Mean (SD) | 11.5 (16.3) | | 5.8 (11.7) | | 7.5 (20.2) | | 6.3 (10.9) | | 8.8 (16.3) | | 0.0 (-) | | - | | - | | - | | 0.0 (NE) |  |
|  | Min–Max | 0.0–50.0 | | 0.0–40.0 | | 0.0–80.0 | | 0.0–30.0 | | 0.0–50.0 | | 0.0–0.0 | | - | | - | | - | | 0.0–0.0 |  |
| Percentage of overall classroom impairment due to haemophilia, % | | | | | | | | | | | | | | | | | | | | | |
| Number of subjects | | 20 | | 19 | | 16 | | 16 | | 16 | | 0 | | 0 | | 0 | | 0 | | 1 |  |
|  | Mean (SD) | 12.1 (16.1) | | 7.5 (14.0) | | 11.7 (27.7) | | 8.2 (13.9) | | 12.2 (17.6) | | - | | - | | - | | - | | 0.0 (NE) |  |
|  | Min–Max | 0.0–50.0 | | 0.0–52.0 | | 0.0–84.0 | | 0.0–41.0 | | 0.0–50.0 | | - | | - | | - | | - | | 0.0–0.0 |  |
| Percentage of activity impairment due to haemophilia, % | | | | | | | | | | | | | | | | | | | | | |
| Number of subjects | | 80 | | 75 | | 73 | | 67 | | 66 | | 20 | | 20 | | 18 | | 19 | | 15 |  |
|  | Mean (SD) | 18.6 (20.6) | | 20.8 (25.9) | | 18.6 (23.9) | | 18.7 (22.3) | | 16.8 (21.0) | | 34.0 (31.5) | | 29.5 (29.8) | | 27.8 (34.6) | | 35.8 (36.4) | | 31.3 (29.0) |  |
|  | Min–Max | 0.0–70.0 | | 0.0–100.0 | | 0.0–100.0 | | 0.0–100.0 | | 0.0–80.0 | | 0.0–100.0 | | 0.0–100.0 | | 0.0–100.0 | | 0.0–100.0 | | 0.0–80.0 |  |

NE, not evaluable; SD, standard deviation

**Table S4.** Subgroup analysis for WPAI+CIQ:HS scores between participants with or without bleeding prior to study entry for participants aged ≥6 years

|  | | **History of no bleeding** | | | | | **History of bleeding** | | | | |
| --- | --- | --- | --- | --- | --- | --- | --- | --- | --- | --- | --- |
|  |  | Week 1 | Week 25 | Week 49 | Week 73 | Week 97 | Week 1 | Week 25 | Week 49 | Week 73 | Week 97 |
| Percentage of work time missed due to haemophilia, % | | | | | | | | | | | |
|  | Number of subjects | 22 | 16 | 20 | 20 | 16 | 37 | 36 | 32 | 32 | 30 |
|  | Mean (SD) | 3.0 (7.7) | 2.1 (6.1) | 0.0 (0.0) | 0.2 (0.9) | 8.1 (25.0) | 0.1 (0.4) | 1.4 (4.7) | 1.4 (7.1) | 3.0 (11.3) | 5.1 (18.7) |
|  | Min–Max | 0.0–28.0 | 0.0–22.9 | 0.0–0.0 | 0.0–4.0 | 0.0–100.0 | 0.0–2.4 | 0.0–25.0 | 0.0–40.0 | 0.0–60.0 | 0.0–100.0 |
| Percentage of impairment while working due to haemophilia, % | | | | | | | | | | | |
|  | Number of subjects | 22 | 16 | 20 | 20 | 16 | 37 | 36 | 33 | 33 | 30 |
|  | Mean (SD) | 9.5 (13.6) | 11.3 (20.6) | 4.5 (7.6) | 9.0 (12.5) | 7.5 (9.3) | 14.9 (19.9) | 13.9 (19.9) | 16.4 (26.3) | 16.7 (20.7) | 16.7 (20.9) |
|  | Min–Max | 0.0–50.0 | 0.0–80.0 | 0.0–20.0 | 0.0–40.0 | 0.0–20.0 | 0.0–80.0 | 0.0–80.0 | 0.0–100.0 | 0.0–80.0 | 0.0–70.0 |
| Percentage of overall work impairment due to haemophilia, % | | | | | | | | | | | |
|  | Number of subjects | 22 | 16 | 20 | 20 | 16 | 37 | 36 | 32 | 32 | 30 |
|  | Mean (SD) | 11.5 (17.6) | 12.4 (22.3) | 4.5 (7.6) | 9.1 (12.8) | 14.2 (25.2) | 14.9 (19.9) | 14.8 (20.7) | 14.2 (22.8) | 18.8 (23.4) | 20.9 (26.2) |
|  | Min–Max | 0.0–58.0 | 0.0–82.0 | 0.0–20.0 | 0.0–42.4 | 0.0–100.0 | 0.0–80.0 | 0.0–80.0 | 0.0–88.0 | 0.0–84.0 | 0.0–100.0 |
| Percentage of class time missed due to haemophilia, % | | | | | | | | | | | |
|  | Number of subjects | 11 | 11 | 7 | 8 | 7 | 9 | 8 | 9 | 8 | 10 |
|  | Mean (SD) | 1.1 (3.6) | 3.6 (8.1) | 11.4 (30.2) | 4.3 (12.2) | 5.7 (15.1) | 0.0 (0.0) | 0.0 (0.0) | 0.4 (1.1) | 0.0 (0.0) | 1.6 (5.0) |
|  | Min–Max | 0.0–12.0 | 0.0–20.0 | 0.0–80.0 | 0.0–34.5 | 0.0–40.0 | 0.0–0.0 | 0.0–0.0 | 0.0–3.3 | 0.0–0.0 | 0.0–15.8 |
| Percentage of impairment in the classroom due to haemophilia, % | | | | | | | | | | | |
|  | Number of subjects | 11 | 11 | 7 | 8 | 7 | 10 | 8 | 9 | 8 | 10 |
|  | Mean (SD) | 7.3 (11.0) | 10.0 (14.1) | 2.9 (7.6) | 7.5 (11.6) | 2.9 (4.9) | 15.0 (20.1) | 0.0 (0.0) | 11.1 (26.2) | 5.0 (10.7) | 12.0 (19.9) |
|  | Min–Max | 0.0–30.0 | 0.0–40.0 | 0.0–20.0 | 0.0–30.0 | 0.0–10.0 | 0.0–50.0 | 0.0–0.0 | 0.0–80.0 | 0.0–30.0 | 0.0–50.0 |
| Percentage of overall classroom impairment due to haemophilia, % | | | | | | | | | | | |
|  | Number of subjects | 11 | 11 | 7 | 8 | 7 | 9 | 8 | 9 | 8 | 10 |
|  | Mean (SD) | 8.4 (10.8) | 12.9 (16.6) | 12.0 (31.7) | 11.4 (16.7) | 8.6 (14.6) | 16.7 (20.6) | 0.0 (0.0) | 11.4 (26.2) | 5.0 (10.7) | 13.6 (19.5) |
|  | Min–Max | 0.0–30.0 | 0.0–52.0 | 0.0–84.0 | 0.0–41.0 | 0.0–40.0 | 0.0–50.0 | 0.0–0.0 | 0.0–80.0 | 0.0–30.0 | 0.0–50.0 |
| Percentage of activity impairment due to haemophilia, % | | | | | | | | | | | |
|  | Number of subjects | 38 | 32 | 33 | 31 | 28 | 62 | 63 | 58 | 55 | 53 |
|  | Mean (SD) | 14.7 (17.7) | 18.1 (22.8) | 12.1 (15.6) | 11.6 (13.2) | 11.8 (17.0) | 26.0 (26.1) | 24.9 (28.6) | 25.2 (30.0) | 28.5 (30.5) | 23.6 (25.0) |
|  | Min–Max | 0.0–70.0 | 0.0–80.0 | 0.0–70.0 | 0.0–50.0 | 0.0–70.0 | 0.0–100.0 | 0.0–100.0 | 0.0–100.0 | 0.0–100.0 | 0.0–80.0 |

SD, standard deviation

**Table S5.** Subgroup analysis for changes in quality of daily life questionnaire outcomes from baseline, completed by participants

|  |  | **Previous factor treatment** | | | | **Disease severity** | | | | **Target joints at baseline** | | | | **History of bleeding** | | | | |
| --- | --- | --- | --- | --- | --- | --- | --- | --- | --- | --- | --- | --- | --- | --- | --- | --- | --- | --- |
|  |  | Prophylaxis | | On demand | | Severe | | Moderate | | None | | Yes | | None | | Yes | | |
| **Time** | | Week 49 | Week 97 | Week 49 | Week 97 | Week 49 | Week 97 | Week 49 | Week 97 | Week 49 | Week 97 | Week 49 | Week 97 | Week 49 | Week 97 | Week 49 | Week 97 |  |
| **Number of respondents** | | 71 | 63 | 18 | 18 | 72 | 67 | 18 | 15 | 72 | 67 | 18 | 15 | 33 | 29 | 57 | 53 |  |
| **Changes in activity, n (%)** | |  |  |  |  |  |  |  |  |  |  |  |  |  |  |  |  |  |
|  | Increased | 8  (11.3) | 5  (7.9) | 3 (16.7) | 3  (16.7) | 7  (9.7) | 5 (7.5) | 4  (22.2) | 3  (20.0) | 8 (11.1) | 6  (9.0) | 3  (16.7) | 2 (13.3) | 4 (12.1) | 3 (10.3) | 7 (12.3) | 5 (9.4) |  |
|  | Slightly increased | 7  (9.9) | 13 (20.6) | 0  (0.0) | 5  (27.8) | 6  (8.3) | 15  (22.4) | 1  (5.6) | 3  (20.0) | 6  (8.3) | 14  (20.9) | 1  (5.6) | 4 (26.7) | 3  (9.1) | 7 (24.1) | 4  (7.0) | 11 (20.8) |  |
|  | No change | 51  (71.8) | 43 (68.3) | 13  (72.2) | 10  (55.6) | 52  (72.2) | 45  (67.2) | 13  (72.2) | 9  (60.0) | 54  (75.0) | 46  (68.7) | 11  (61.1) | 8 (53.3) | 25 (75.8) | 19 (65.5) | 40  (70.2) | 35 (66.0) |  |
|  | Slightly decreased | 4  (5.6) | 2  (3.2) | 2  (11.1) | 0  (0.0) | 6  (8.3) | 2  (3.0) | 0  (0.0) | 0  (0.0) | 3  (4.2) | 1  (1.5) | 3  (16.7) | 1  (6.7) | 1  (3.0) | 0  (0.0) | 5  (8.8) | 2 (3.8) |  |
|  | Decreased | 1  (1.4) | 0  (0.0) | 0  (0.0) | 0  (0.0) | 1  (1.4) | 0  (0.0) | 0  (0.0) | 0  (0.0) | 1  (1.4) | 0  (0.0) | 0  (0.0) | 0  (0.0) | 0  (0.0) | 0  (0.0) | 1 (1.8) | 0 (0.0) |  |
| **Changes in exercise frequency, n (%)** | |  |  |  |  |  |  |  |  |  |  |  |  |  |  |  |  |  |
|  | Increased | 6  (8.5) | 6  (9.5) | 2  (11.1) | 2  (11.1) | 6  (8.3) | 5  (7.5) | 2  (11.1) | 3 (20.0) | 6  (8.3) | 7  (10.4) | 2  (11.1) | 1  (6.7) | 3  (9.1) | 4 (13.8) | 5  (8.8) | 4 (7.5) |  |
|  | Slightly increased | 6  (8.5) | 12 (19.0) | 2  (11.1) | 6  (33.3) | 8  (11.1) | 15  (22.4) | 0  (0.0) | 3 (20.0) | 7  (9.7) | 14  (20.9) | 1  (5.6) | 4 (26.7) | 3  (9.1) | 6 (20.7) | 5  (8.8) | 12 (22.6) |  |
|  | No change | 56  (78.9) | 39 (61.9) | 13  (72.2) | 8  (44.4) | 54  (75.0) | 40  (59.7) | 16  (88.9) | 8 (53.3) | 56  (77.8) | 40  (59.7) | 14  (77.8) | 8 (53.3) | 25 (75.8) | 17 (58.6) | 45  (78.9) | 31 (58.5) |  |
|  | Slightly decreased | 1  (1.4) | 4 (6.3) | 1  (5.6) | 2  (11.1) | 2  (2.8) | 5  (7.5) | 0  (0.0) | 1  (6.7) | 1  (1.4) | 6  (9.0) | 1  (5.6) | 0  (0.0) | 1  (3.0) | 2  (6.9) | 1  (1.8) | 4 (7.5) |  |
|  | Decreased | 2  (2.8) | 2 (3.2) | 0  (0.0) | 0  (0.0) | 2  (2.8) | 2  (3.0) | 0  (0.0) | 0  (0.0) | 2  (2.8) | 0  (0.0) | 0  (0.0) | 2 (13.3) | 1  (3.0) | 0  (0.0) | 1  (1.8) | 2 (3.8) |  |
| **Change in motivation for work, etc., n (%)** | |  |  |  |  |  |  |  |  |  |  |  |  |  |  |  |  |  |
|  | Increased | 5  (7.0) | 8 (12.7) | 1  (5.6) | 3  (16.7) | 5  (6.9) | 6  (9.0) | 1  (5.6) | 5 (33.3) | 4  (5.6) | 9  (13.4) | 2  (11.1) | 2 (13.3) | 4  (12.1) | 5 (17.2) | 2  (3.5) | 6 (11.3) |  |
|  | Slightly increased | 11  (15.5) | 8 (12.7) | 6  (33.3) | 5  (27.8) | 13  (18.1) | 12  (17.9) | 4  (22.2) | 1  (6.7) | 11  (15.3) | 7  (10.4) | 6  (33.3) | 6 (40.0) | 4  (12.1) | 4 (13.8) | 13  (22.8) | 9 (17.0) |  |
|  | No change | 50  (70.4) | 47 (74.6) | 11  (61.1) | 10  (55.6) | 50  (69.4) | 49  (73.1) | 12  (66.7) | 9 (60.0) | 54  (75.0) | 51  (76.1) | 8  (44.4) | 7 (46.7) | 23  (69.7) | 20 (69.0) | 39  (68.4) | 38 (71.7) |  |
|  | Slightly decreased | 4  (5.6) | 0 (0.0) | 0  (0.0) | 0  (0.0) | 3  (4.2) | 0  (0.0) | 1  (5.6) | 0  (0.0) | 3  (4.2) | 0  (0.0) | 1  (5.6) | 0  (0.0) | 2  (6.1) | 0  (0.0) | 2  (3.5) | 0 (0.0) |  |
|  | Decreased | 1  (1.4) | 0 (0.0) | 0  (0.0) | 0  (0.0) | 1  (1.4) | 0  (0.0) | 0  (0.0) | 0  (0.0) | 0  (0.0) | 0  (0.0) | 1  (5.6) | 0  (0.0) | 0  (0.0) | 0  (0.0) | 1  (1.8) | 0 (0.0) |  |
| **Change in anxiety about bleeding, n (%)** | |  |  |  |  |  |  |  |  |  |  |  |  |  |  |  |  |  |
|  | Increased | 3  (4.2) | 1  (1.6) | 0  (0.0) | 0  (0.0) | 3  (4.2) | 1  (1.5) | 0  (0.0) | 0  (0.0) | 3  (4.2) | 1  (1.5) | 0  (0.0) | 0  (0.0) | 0  (0.0) | 0  (0.0) | 3  (5.3) | 1 (1.9) |  |
|  | Slightly increased | 6  (8.5) | 9  (14.3) | 0  (0.0) | 0  (0.0) | 5  (6.9) | 9  (13.4) | 1  (5.6) | 0  (0.0) | 4  (5.6) | 7  (10.4) | 2  (11.1) | 2 (13.3) | 2  (6.1) | 3 (10.3) | 4  (7.0) | 6 (11.3) |  |
|  | No change | 27  (38.0) | 16 (25.4) | 4  (22.2) | 4  (22.2) | 26  (36.1) | 18  (26.9) | 5  (27.8) | 3 (20.0) | 29  (40.3) | 20  (29.9) | 2  (11.1) | 1  (6.7) | 14  (42.4) | 7 (24.1) | 17  (29.8) | 14 (26.4) |  |
|  | Slightly decreased | 18  (25.4) | 22  (34.9) | 7  (38.9) | 7  (38.9) | 18  (25.0) | 21  (31.3) | 8  (44.4) | 8 (53.3) | 20  (27.8) | 24  (35.8) | 6  (33.3) | 5 (33.3) | 8  (24.2) | 10 (34.5) | 18  (31.6) | 19 (35.8) |  |
|  | Decreased | 17  (23.9) | 15  (23.8) | 7  (38.9) | 7  (38.9) | 20 (27.8) | 18  (26.9) | 4  (22.2) | 4 (26.7) | 16  (22.2) | 15  (22.4) | 8  (44.4) | 7 (46.7) | 9  (27.3) | 9 (31.0) | 15  (26.3) | 13 (24.5) |  |
| **Participation in physical education, n (%)** | |  |  |  |  |  |  |  |  |  |  |  |  |  |  |  |  |  |
|  | Number of respondents | 21 | 19 | 3 | 3 | 18 | 18 | 6 | 4 | 24 | 22 | 0 | 0 | 11 | 10 | 13 | 12 |  |
|  | Participation in all | 13  (61.9) | 15  (78.9) | 3  (100.0) | 3  (100.0) | 14  (77.8) | 15  (83.3) | 2  (33.3) | 3 (75.0) | 16  (66.7) | 18  (81.8) | - | - | 6  (54.5) | 9 (90.0) | 10  (76.9) | 9 (75.0) |  |
|  | Participate according to the event | 5  (23.8) | 3  (15.8) | 0  (0.0) | 0  (0.0) | 2  (11.1) | 2  (11.1) | 3  (50.0) | 1 (25.0) | 5  (20.8) | 3  (13.6) | - | - | 3  (27.3) | 0  (0.0) | 2  (15.4) | 3 (25.0) |  |
|  | Not participating at all | 3  (14.3) | 1  (5.3) | 0  (0.0) | 0  (0.0) | 2  (11.1) | 1  (5.6) | 1  (16.7) | 0  (0.0) | 3  (12.5) | 1  (4.5) | - | - | 2  (18.2) | 1 (10.0) | 1  (7.7) | 0 (0.0) |  |

**Table S6.** Individual participant comments from the daily life questionnaire

| **Comparison between before and after emicizumab initiation** |
| --- |
| I have done more than healthy sports to begin with, so there is no change to the above questions, but I felt more comfortable with the bleeding after starting emicizumab. |
| The patient could not inject blood products, except when playing golf or standing for a long time. If I miss my right elbow supporter, I get internal bleeding and feel anxious all year long! I left it on, but let go of the supporter after emicizumab was administered. I no longer have anxiety about bleeding in the right arm. |
| I am mentally more at ease now that I am taking medications less frequently |
| If I could have used it at a younger age, my life would have been different. It is too late after the joint deforms. |
| Before using emicizumab, the patient always brought the drug when staying overnight, etc., but now the patient does not bring it. The range of activities such as travel may be expanded without concern about bleeding, hemostasis, etc. |
| Feeling less anxious about being out of the drug |
| I feel better now that I have fewer injections |
| Almost no discomfort due to haemorrhage |
| The frequency of injections was 3 times a week in the past, but it became about 2 times a month, and it made life easier. |
| Bleeding anxiety decreased |
| Joint haemorrhage almost disappeared, and the patient became mentally comfortable. |
| My elbows are easier |
| (1) From the start, the patient usually carried out work. (2) Walking was started because haemorrhage disappeared. (3) Positive feelings about anything increased. (4) Anxiety almost disappeared. |
| Almost never anxious |
| I am less concerned about movement at work and at home. The patient started to receive emicizumab and bleeding disappeared, so it has been very helpful. |
| Bleeding decreased. I feel that the reduction in the frequency of injections is the biggest thing. |
| The patient was able to continue walking almost every day. |
| Since bleeding in the elbow and ankle joints disappeared, the amount of daily activity increased. As a result, weight loss began, and a virtuous cycle began in which it became even easier to be active. Bleeding in the knee joint occurred occasionally, but the patient was able to start muscle training around the joint, and the pain when walking is disappearing. |
| Since the opportunities for administration have greatly decreased, I am less likely to worry about drugs. |
| The patient did not stop until now even after cutting her hand, but the patient stopped immediately as soon as she did not stop. |
| Until now, the patient exercised with anxiety, but after the start of emicizumab, the patient became able to exercise without feeling anxiety. I feel positive, probably because of the above sense of security. |
| The interval of injection was prolonged, and subcutaneous injection reduced mental burden. |

Participant comments are translated from Japanese.

**Table S7.** Subgroup analysis for changes in quality of daily life questionnaire outcomes from baseline, completed by caregivers

|  |  | **Previous factor treatment** | | | | **Disease severity** | | | | **Target joints at baseline** | | | | **History of bleeding** | | | | |
| --- | --- | --- | --- | --- | --- | --- | --- | --- | --- | --- | --- | --- | --- | --- | --- | --- | --- | --- |
|  |  | Prophylaxis | | On demand | | Severe | | Moderate | | None | | Yes | | None | | Yes | | |
| **Time** | | Week 49 | Week 97 | Week 49 | Week 97 | Week 49 | Week 97 | Week 49 | Week 97 | Week 49 | Week 97 | Week 49 | Week 97 | Week 49 | Week 97 | Week 49 | Week 97 |  |
| **Number of respondents** | | 22 | 21 | 10 | 10 | 30 | 29 | 7 | 6 | 35 | 34 | 2 | 1 | 16 | 14 | 21 | 21 |  |
| **Relationship to patient, n (%)** | |  |  |  |  |  |  |  |  |  |  |  |  |  |  |  |  |  |
|  | Mother | 17 (77.3) | 16 (76.2) | 8 (80.0) | 9 (90.0) | 25 (83.3) | 25 (86.2) | 5 (71.4) | 4 (66.7) | 29 (82.9) | 28 (82.4) | 1 (50.0) | 1 (100.0) | 12 (75.0) | 10 (71.4) | 18 (85.7) | 19 (90.5) |  |
|  | Father | 4 (18.2) | 3 (14.3) | 2 (20.0) | 1 (10.0) | 4 (13.3) | 2 (6.9) | 2 (28.6) | 2 (33.3) | 5 (14.3) | 4 (11.8) | 1 (50.0) | 0 (0.0) | 4 (25.0) | 3 (21.4) | 2 (9.5) | 1 (4.8) |  |
|  | Spouse | 1 (4.5) | 1 (4.8) | 0 (0.0) | 0 (0.0) | 1 (3.3) | 1 (3.4) | 0 (0.0) | 0 (0.0) | 1 (2.9) | 1 (2.9) | 0 (0.0) | 0 (0.0) | 0 (0.0) | 0 (0.0) | 1 (4.8) | 1 (4.8) |  |
|  | Other | 0 (0.0) | 1 (4.8) | 0 (0.0) | 0 (0.0) | 0 (0.0) | 1 (3.4) | 0 (0.0) | 0 (0.0) | 0 (0.0) | 1 (2.9) | 0 (0.0) | 0 (0.0) | 0 (0.0) | 1 (7.1) | 0 (0.0) | 0 (0.0) |  |
| **Changes in activity, n (%)** | |  |  |  |  |  |  |  |  |  |  |  |  |  |  |  |  |  |
|  | Increased | 1 (4.5) | 4 (19.0) | 4 (40.0) | 3 (30.0) | 4 (13.3) | 8 (27.6) | 1 (14.3) | 0 (0.0) | 5 (14.3) | 8 (23.5) | 0 (0.0) | 0 (0.0) | 2 (12.5) | 3 (21.4) | 3 (14.3) | 5 (23.8) |  |
|  | Slightly increased | 4 (18.2) | 4 (19.0) | 0 (0.0) | 2 (20.0) | 4 (13.3) | 4 (13.8) | 1 (14.3) | 2 (33.3) | 5 (14.3) | 6 (17.6) | 0 (0.0) | 0 (0.0) | 3 (18.8) | 2 (14.3) | 2 (9.5) | 4 (19.0) |  |
|  | No change | 15 (68.2) | 13 (61.9) | 6 (60.0) | 5 (50.0) | 20 (66.7) | 17 (58.6) | 5 (71.4) | 4 (66.7) | 23 (65.7) | 20 (58.8) | 2 (100.0) | 1 (100.0) | 10 (62.5) | 9 (64.3) | 15 (71.4) | 12 (57.1) |  |
|  | Slightly decreased | 0 (0.0) | 0 (0.0) | 0 (0.0) | 0 (0.0) | 0 (0.0) | 0 (0.0) | 0 (0.0) | 0 (0.0) | 0 (0.0) | 0 (0.0) | 0 (0.0) | 0 (0.0) | 0 (0.0) | 0 (0.0) | 0 (0.0) | 0 (0.0) |  |
|  | Decreased | 2 (9.1) | 0 (0.0) | 0 (0.0) | 0 (0.0) | 2 (6.7) | 0 (0.0) | 0 (0.0) | 0 (0.0) | 2 (5.7) | 0 (0.0) | 0 (0.0) | 0 (0.0) | 1 (6.3) | 0 (0.0) | 1 (4.8) | 0 (0.0) |  |
| **Changes in work and other initiatives, n (%)** | |  |  |  |  |  |  |  |  |  |  |  |  |  |  |  |  |  |
|  | Increased | 3 (13.6) | 3 (14.3) | 4 (40.0) | 3 (30.0) | 5 (16.7) | 6 (20.7) | 2 (28.6) | 0 (0.0) | 7 (20.0) | 6 (17.6) | 0 (0.0) | 0 (0.0) | 4 (25.0) | 3 (21.4) | 3 (14.3) | 3 (14.3) |  |
|  | Slightly increased | 1 (4.5) | 2 (9.5) | 0 (0.0) | 1 (10.0) | 1 (3.3) | 3 (10.3) | 0 (0.0) | 0 (0.0) | 1 (2.9) | 3 (8.8) | 0 (0.0) | 0 (0.0) | 0 (0.0) | 3 (21.4) | 1 (4.8) | 0 (0.0) |  |
|  | No change | 17 (77.3) | 16 (76.2) | 6 (60.0) | 5 (50.0) | 23 (76.7) | 20 (69.0) | 5 (71.4) | 5 (83.3) | 26 (74.3) | 24 (70.6) | 2 (100.0) | 1 (100.0) | 12 (75.0) | 7 (50.0) | 16 (76.2) | 18 (85.7) |  |
|  | Slightly decreased | 1 (4.5) | 0 (0.0) | 0 (0.0) | 1 (10.0) | 1 (3.3) | 0 (0.0) | 0 (0.0) | 1 (16.7) | 1 (2.9) | 1 (2.9) | 0 (0.0) | 0 (0.0) | 0 (0.0) | 1 (7.1) | 1 (4.8) | 0 (0.0) |  |
|  | Decreased | 0 (0.0) | 0 (0.0) | 0 (0.0) | 0 (0.0) | 0 (0.0) | 0 (0.0) | 0 (0.0) | 0 (0.0) | 0 (0.0) | 0 (0.0) | 0 (0.0) | 0 (0.0) | 0 (0.0) | 0 (0.0) | 0 (0.0) | 0 (0.0) |  |
| **Change in anxiety about bleeding, n (%)** | |  |  |  |  |  |  |  |  |  |  |  |  |  |  |  |  |  |
|  | Increased | 2 (9.1) | 0 (0.0) | 0 (0.0) | 0 (0.0) | 1 (3.3) | 0 (0.0) | 1 (14.3) | 0 (0.0) | 2 (5.7) | 0 (0.0) | 0 (0.0) | 0 (0.0) | 1 (6.3) | 0 (0.0) | 1 (4.8) | 0 (0.0) |  |
|  | Slightly increased | 0 (0.0) | 2 (9.5) | 1 (10.0) | 0 (0.0) | 1 (3.3) | 2 (6.9) | 0 (0.0) | 0 (0.0) | 1 (2.9) | 2 (5.9) | 0 (0.0) | 0 (0.0) | 1 (6.3) | 0 (0.0) | 0 (0.0) | 2 (9.5) |  |
|  | No change | 6 (27.3) | 8 (38.1) | 1 (10.0) | 3 (30.0) | 8 (26.7) | 11 (37.9) | 1 (14.3) | 2 (33.3) | 9 (25.7) | 13 (38.2) | 0 (0.0) | 0 (0.0) | 3 (18.8) | 6 (42.9) | 6 (28.6) | 7 (33.3) |  |
|  | Slightly decreased | 9 (40.9) | 7 (33.3) | 5 (50.0) | 3 (30.0) | 13 (43.3) | 9 (31.0) | 4 (57.1) | 3 (50.0) | 16 (45.7) | 11 (32.4) | 1 (50.0) | 1 (100.0) | 5 (31.3) | 4 (28.6) | 12 (57.1) | 8 (38.1) |  |
|  | Decreased | 5 (22.7) | 4 (19.0) | 3 (30.0) | 4 (40.0) | 7 (23.3) | 7 (24.1) | 1 (14.3) | 1 (16.7) | 7 (20.0) | 8 (23.5) | 1 (50.0) | 0 (0.0) | 6 (37.5) | 4 (28.6) | 2 (9.5) | 4 (19.0) |  |

**Table S8.** Individual caregiver comments from the daily life questionnaire

| **Relationship to patient** | **Patients compared to prior to starting emicizumab** |
| --- | --- |
| Mother | Since my child has grown and I have almost no concerns about bleeding, my job has changed from a part-time job to a full-time job. It became easier to inject once a week, and I had no anxiety. However, I sometimes forget that I have haemophilia. |
| Mother | For several months after birth, my child did not move much, so I tried to play in bed as much as possible, and I did not have any problems. However, after I started to inject emicizumab, my child became able to move more freely. I now have less concern about bleeding and can have my brother play in the same space as my brother. |
| Mother | As children get older, they become more active, and when they come back from kindergarten, etc., they sometimes make blue azae. Doctors thoroughly report the background, and the patients themselves tell us, for example, “You hit me on the stairs.” Fortunately, there were no major injuries, but I still feel anxious all the time. |
| Mother | Since the patient is a preschool child, there is no change in activities, but anxiety about injuries as a parent is alleviated. Even when mild subcutaneous bleeding occurs, I feel that it heals quickly. |
| Mother | When a wound was formed or injected, it quickly became swollen and the blood did not go away, but after emicizumab was injected, the frequency decreased and I think I can play as much as I want. |
| Mother | After I started using emicizumab, the frequency of bruising obviously decreased, and I feel like it does not take long to bleed. I started a gymnastic class, and I am less concerned about restricted exercise. |
| Mother | Since the patient is still 2 years old, I do not know the criteria for the range of behaviour. Compared to the older child, the patient is active, and bleeding is sometimes suspected, but probably because the patient is receiving emicizumab, it is necessary to stop bleeding immediately. It would be nice if the patient could continue playing and having fun with friends on his own will. |
| Mother | He became able to live his life without worrying about school events and daily activities. |
| Mother | For the first time, I was able to enjoy a long vacation without worrying about injections or injuries. |
| Mother | In the past, I was often careful not to bleed when I was living in preschool or at home, but after I began to receive injections, I was able to freely play with myself, which made me very comfortable emotionally. |
| Mother | The constant bruises on the legs and arms have disappeared, and joint swelling has greatly decreased. I can now make them stop doing things. |
| Mother | Now, I live far from the way I had imagined, in a good sense, and I can live my life without any major anxiety. I appreciate it. |
| Mother | Before I started emicizumab, I had a tendency to restrict my actions, regardless of what I could do if I fell and hit myself. But now, I can get used to not restricting myself too much, saying that if I hit myself, I won't rush it or it's OK. |
| Father | Before I started emicizumab I was always anxious about the risk of bleeding, but when I move around actively with emicizumab I don't feel anxious. In addition, in the past, patients were urgently transported to the hospital due to sudden haemorrhage and were injected with the drug product, but now, there are none, and it has become very easy to spend my daily life. |
| Mother | Even when bleeding occurred after the start of treatment, it stopped immediately and the bruises also decreased, so I felt the effects of treatment. Thanks to this, I was able to encourage them to try various things like other children! |
| Mother | At first, I was anxious, but even when I got bigger and started to move more actively, there was no noticeable bleeding, so I am at ease to some extent. |
| Mother | I was able to expand my range of activities by giving injections. Slides, etc. |
| Mother | Since it is still small, there is not much change in my life. I started emicizumab injection without causing major bleeding. Since I have not been injured yet, I do not feel any effect, but I feel a sense of security that I am receiving emicizumab. |
| Mother | Administration of emicizumab slightly reduced the child's anxiety about exercise. |

Caregiver comments are translated from Japanese
